# Googling and health anxiety in older age—A case study of CBT incorporating a single case experimental design

**DOI:** 10.1002/ccr3.9316

**Published:** 2024-08-10

**Authors:** Sean Hill, Daniel Watts

**Affiliations:** ^1^ The Oxford Institute of Clinical Psychology Training and Research University of Oxford Oxford UK; ^2^ Oxford Health NHS Foundation Trust Oxford UK

**Keywords:** cognitive‐behavioral therapy, cyberchondria, health anxiety, mental health, older adults

## Abstract

The use of online search engines to “google” health‐related information is common in health anxiety, and requires careful consideration within psychological treatment. Its nature in older adult populations is less closely understood. This report demonstrates the reduction in googling frequency using cognitive‐behavioral therapy in an 83‐year‐old female with health anxiety.

## Graphical abstract

Vicious flower maintenance formulation of client's health anxiety, adapted from Salkovskis et al. (*Brief Treat Crisis Interv*, 2003, 3, 353).

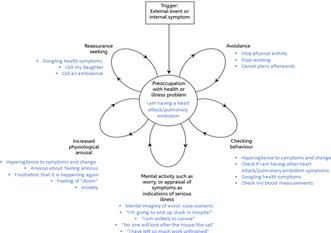

## 1 INTRODUCTION

Health anxiety is defined by the International Classification of Diseases—ICD‐11 (6B23^1^) as a preoccupation and/or fear of experiencing serious or life‐threatening illnesses. These fears are hallmarked by recurrent hypervigilance about health symptoms, catastrophic interpretation of their cause, seeking reassurance and/or psychological or physical safety (e.g. through medical appointments or online symptom searching), and/or avoidance behaviors, such as unattendance at medical appointments or restricting activity. These features lead to significant distress and impairment to interpersonal, familial, educational occupational, and other important areas of functioning.

Etiology of health anxiety is unclear and largely heterogenous, but risk appears to increase following significant health events (e.g. a heart attack), presence of physical health conditions, traumatic experiences involving health, death of loved ones, low socioeconomic status, and comorbid or premorbid anxiety disorders.^2^

The prevalence of health anxiety increases with age, and is more readily associated with age than any other anxiety disorder.^3^, ^4^, ^5^ This in part may be explained by associations between age and health experiences, leading to newly‐onset health anxiety in previously non‐anxious individuals as well as re‐emergence of previous anxiety.^6^ As people age, they develop cumulative experiences related to ill health, not limited to death of family and friends, chronic health conditions, and age‐related frailty. By older adult age, these often effectuate salience to mortality and health,^3^ which may include expectations of imminent deterioration and death.^7^ These coincide with socioeconomic vulnerabilities, such as social isolation, reductions in independence, limited engagement in physical activity, and reduced social and financial resources.^8^

### 1.1 Googling and health anxiety

The use of online search engines, such as Google, to obtain health‐related information is a phenomenon of emerging interest in health anxiety.^9^, ^10^ One study indicated over half of a general population sample use Google as their first source of information about their own health,^11^ and many people believe information from Google is highly accurate.^12^ Despite this, online information can have low reliability and be subject to misinterpretation by users.^9^

The nature and function of “googling” is unclear and likely heterogenous. Googling symptoms could be interpreted as a form of rumination that additionally involves access to health‐related information which is unduly interpreted as valid and medically endorsed.^13^ It might also resemble reassurance‐seeking through the searching of diagnoses specific to symptoms being experienced.^9^

Cognitive behavioral therapy (CBT) is a recommended treatment for anxiety disorders within current NICE guidance; there is no specific guidance for health anxiety. Calls for such guidance have been made due to increasing prevalence of health anxiety, due potentially to the rise in online information about health.^14^ CBT combines manualised treatment protocols with personalized adaptations, designed collaboratively with the client, allowing the treatment to be uniquely individualized. Within older adults, evidence of efficacy for CBT in health anxiety is limited^15^ with further case reports indicated for exploring treatment presentation and adaptation.

The present report details the delivery of CBT for an older adult with health anxiety, incorporating googling as a specific maintenance variable. A single case experimental design was conducted to explore how CBT may affect googling frequency.

## 2 CASE REPORT

Evie was a 73‐year‐old White British female. From the age of 65, she was increasingly debilitated with preoccupation about her physical health. This led to avoidance of activities that may cause physical strain, rumination about health, hypervigilance to health changes with catastrophic interpretations of their cause, and a negative view of herself due to the impact these were having on her life. She had become increasingly withdrawn; Evie regularly contacted people via phone but rarely visited them, feeling unable to due to health‐related anxiety. She regularly used online search engines, such as Google, to find health‐related information, which she stated would keep her awake at night and lead to heightened anxiety about the potential presence of conditions she “googled.”

Evie lived alone following separation from her husband nearly 35 years prior, and lived close to her daughter, her daughter's husband, and numerous friends of a similar age. She had no grandchildren due to filial infertility. Many of Evie's childhood friends had either died or were suffering from debilitating physical and/or mental illnesses. Evie suffered a pulmonary embolism in 2020, and had numerous health conditions including cardiovascular disease, osteoporosis, broken wrists, hip replacements, glaucoma, and cataracts. From this, she felt a sense of inevitable “doom” as she increasingly identified with older age and cumulative health experiences.

Evie self‐referred to an NHS Talking Therapies service due to a belief that she was “overly self‐limiting” various aspects of life due to health beliefs. She was offered a course of CBT following a telephone assessment, and 7 weeks on the service's waiting list.

### 2.1 Case history and examination

Evie reported health anxiety first presenting in her childhood. Aged 8, she supported her younger sister who fell severely ill with pneumonia and survived. Shortly after, Evie lived with her grandmother, who died suddenly and unexpectedly when Evie was 10 from an unknown illness. At age 11, Evie fell ill herself with an unknown debilitating illness, and was hospitalized for roughly 3 weeks, geographically far from her familial home. She had no visitors and was given unprecise information about her condition, prognosis, and when she could return home. She described the experience as traumatic and feared she was going to die. Not wanting others to feel sorry for her, Evie “put on a brave face” and described presenting to be unbothered by her lack of visitors. She hypothesized that this instilled beliefs that if she was to become seriously ill again, she would be unsupported and “suffer alone.” Following discharge, this experience instilled catastrophic beliefs about developing future illness, increased salience to her physical state, and belief that she can become severely ill at any time, even if currently healthy.

Throughout adolescent and adult life, Evie remained hypervigilant and avoidant to sources of potential contamination: she described regularly finding excuses not to eat meals that had not been cooked by herself or professionals, and being highly avoidant of the presence of others who were unwell. She maintained relatively good health throughout adult life, attributing this partly to her lifetime of hypervigilance and safety‐seeking behaviors.

As she progressed to older age and experience cumulative negative experiences related to health, Evie's preoccupation with her physical increased, which engendered a belief she was likely to experience another significant health event, such as a heart attack or pulmonary embolism. This led to avoidance of important life activities such as engaging in pleasurable outdoor activities, and cleaning her home, due to a fear the exertion required may trigger a catastrophic health event. Her regular “googling” of symptoms maintained these beliefs, and impacted her daily routines and sleep schedule, due to googling for multiple hours at nighttime.

In Evie's childhood home, there was an encyclopedia of medical conditions and symptoms which she took an interest in. Following her traumatic hospital experiences, use of this encyclopedia began to function as reassurance‐seeking as to the potential causes of health symptoms she was experiencing. She reported that this would often lead to her seeing those symptoms in life‐threatening conditions and believe she was suffering from them. As Evie grew older, and with the rise of online search engines, such as Google, Evie increasingly “googled” health symptoms in a similar manner. She believed googling was worse due to the greater volume of information and higher potential to “go down a rabbit hole” of searching conditions extensively.

## 3 METHOD

### 3.1 Psychometric assessment

The Health Anxiety Inventory (HAI), Generalized Anxiety Disorder 7‐item scale (GAD‐7), Patient Health Questionnaire 9‐item scale (PHQ‐9), and Work and Social Adjustment Scale (WSAS) were administered weekly at each session. Health symptom googling was assessed using an idiographic self‐report measure created collaboratively prior to treatment, administered weekly for 3 weeks prior to the start of treatment and, biweekly when treatment commenced. This measure contained two questions: “In the last seven days… (1) How many times did you google health symptoms? (2) On average, how long did you spend googling symptoms?” Answers were multiplied to give a total weekly time.

Frequency of health‐related googling was measured using a single case experiment (AB) design.^16^ Pre‐intervention (Phase A) was the baseline to establish a stable pattern, taken weekly, 3 weeks prior to treatment. Intervention (Phase B) measurements were taken biweekly throughout treatment, chosen over weekly measurement to reduce burden on session time due to completing measures. A 3‐month follow‐up was also completed. Googling frequency was calculated using an idiographic measure constructed collaboratively with the client.

### 3.2 Formulation

Two collaborative formulations providing a cross‐sectional and longitudinal understanding of Evie's health anxiety were constructed. The first model focused on cross‐sectional maintenance mechanisms of health anxiety^17^ and the second used a longitudinal CBT formulation for older adults.^18^ The function of the second formulation was to remain salient to potential complexities related to older age, including physical health conditions, intergenerational linkages, cohort beliefs, role transitions, and sociocultural context.^19^

### 3.3 Cognitive‐behavioral therapy intervention

Evie completed 12 sessions of CBT for health anxiety, in accordance with Salkovskis et al.'s^17^ protocol with additional older adult CBT modifications.^18^ Collaboratively, the following goals were established: (1) reduce symptoms of health anxiety, anxiety and depression as measured by psychometrics; (2) reduce avoidance of activities due to health‐related fears; (3) develop skills and coping strategies related to health anxiety; and (4) reduce the frequency of symptom googling.

#### 3.3.1 Psychoeducation, goal‐setting, rapport building

Sessions 1–3 focused on engagement with the treatment model. Rapport building incorporated voicing differences in age and gender between Evie and the clinician, facilitating these aspects being included appropriately in goal‐setting and formulation. Psychoeducation focused on extending Evie's understanding about health symptom maintenance beyond a traditional medical model, incorporating psychological factors.

#### 3.3.2 Collaborative guided discovery

Formulation development was mostly through Sessions 1–5 but was refined throughout treatment. Guided discovery was facilitated through Socratic questioning, reflections during psychoeducation, mood/thought record homework tasks, and “Theory A/ Theory B” cognitive exercises.

#### 3.3.3 Cognitive intervention

Evie completed a mood/thought record of health anxiety weekly from Session 2. Examples from these records were discussed in sessions to refine formulations and tailor interventions. From Session 6, this was additionally used to elevate mood/reduce depressive symptoms through asking Evie to consider positive qualities and change.

Belief questioning, using downward arrowing and “Theory A/Theory B” exercises, allowed Evie to understand of her own maladaptive self‐protection strategies and healthier alternatives. These exercises engender change through identifying maladaptive beliefs (theory A), examining the evidence for beliefs, consider alternatives (Theory B), and develop more balanced thoughts.

In examples Evie discussed, “Theory A” typically composed of a new health symptom representing a health emergency; “Theory B” composed the alternative explanations for the symptom. Belief in Theory B appeared to elevate most readily using a “responsibility pie” exercise^20^ on Session 7, wherein the percentage likelihood of each explanation being true is added to a pie chart, with the catastrophic “Theory A” explanation added last. This exercise demonstrates that considering alternative possibilities elicits a lower perception of “Theory A” being true than the client initially presumes.

#### 3.3.4 Behavioral intervention

Grounding techniques were practiced in session and as homework to facilitate Evie's ability to disrupt maladaptive behavior patterns, one‐per‐session between Sessions 5 and 10. This included: designing a soothe box with grounding objects; distracting activities Evie enjoyed such as sudoku and codewords; mindfulness‐based grounding exercises.

Behavioral activation functioned to increase mood and reduce avoidance. This constituted Evie focusing on positive aspects within her mood/thought record, activity scheduling at the start of each week, and reflecting on progress.

#### 3.3.5 Relapse prevention

A collaborative safety plan incorporating suicide and personal risk was developed in Session 2 and monitored regularly. Sessions 11 and 12 focused on consolidating Evie's treatment into a worksheet, provided alongside her discharge letter, should symptoms remerge.

## 4 RESULTS

### 4.1 Cross‐sectional formulation

In Evie's initial treatment sessions, her and the clinician described numerous examples of health anxiety which coalesced to two scenario type. The first was when experiencing a new physical symptom, such as pain or dizziness. These examples involved an acute hypervigilance to physical symptoms, and immediate catastrophic thoughts such as “*I am having a heart attack/stroke/pulmonary embolism*.” This resulted in checking for other symptoms associated with that condition, and a positive feedback loop of anxiety and hypervigilance. Evie would then exhibit reassurance‐seeking or safety‐seeking behaviors such as googling or calling an ambulance. This was usually accompanied by imagery of the worst‐case scenario: Evie might visualize herself unconscious being carried into an ambulance, with paramedics and her family seeing the untidy state of her home, and no one being able to feed her cat, leading to it passing away. Evie explained that she felt assuming the worst was positive and protective, as it prepared her for the worst‐case scenario. This anxiety cycle would only subside as symptoms declined, usually 15–30 min after onset. She described avoiding numerous activities she enjoyed for fear of triggering this type of scenario, due to a high belief it represented the onset of catastrophic health events.

The second type of scenario was the onset of ruminative thinking patterns related to health during periods of inactivity, such as while trying to sleep. These thoughts would lead to heightened anxiety and an “irresistible” urge to google health symptoms she was experiencing or had recently experienced, to see if a serious condition could be responsible.

The googling appeared to serve two functions for Evie. First, she described wanting to gain reassurance from googling, although noted that this did not often lead to reductions in anxiety as the googling generally indicated to Evie she was likely suffering from a serious health condition.

The second function appeared to be a parallel and externalized form of rumination. While googling, Evie would read extensive symptom lists of different conditions, and recall times she had recently experienced them. This would act as further evidence she was suffering from a serious illness, leading to further anxiety, and a sense of “doom.” This was disruptive to her sleep and, resultantly, her routine.

**FIGURE 1:**
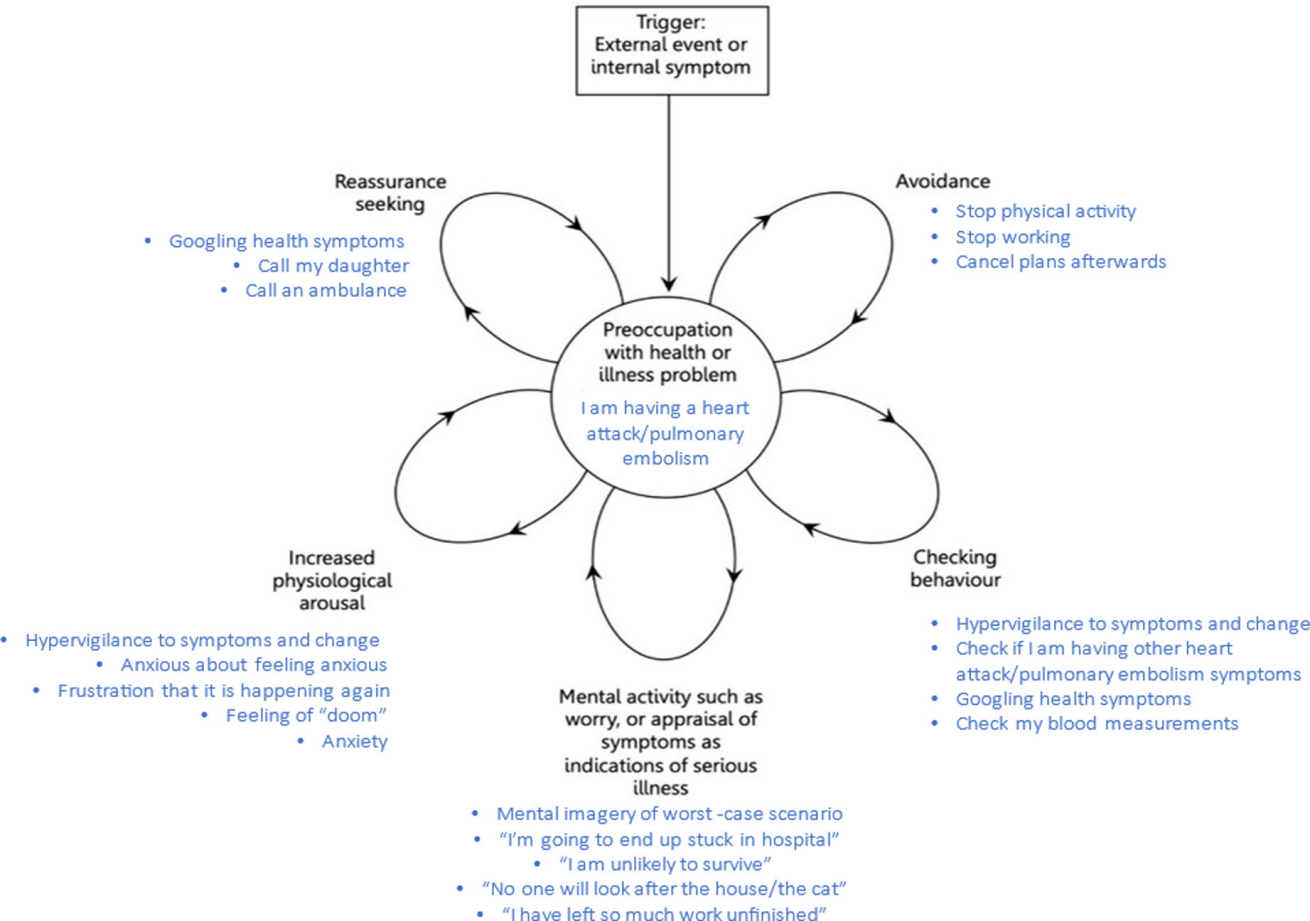
Vicious flower maintenance formulation of Evie's health anxiety, adapted from Salkovskis et al.^17^

These examples constitute maintenance patterns of hypervigilance to physical health changes, avoidance behaviors, symptom checking, emotional arousal and worry. These are illustrated in diagrammatic form using a vicious flower maintenance formulation in Figure 1.^17^

### 4.2 Longitudinal formulation

**FIGURE 2:**
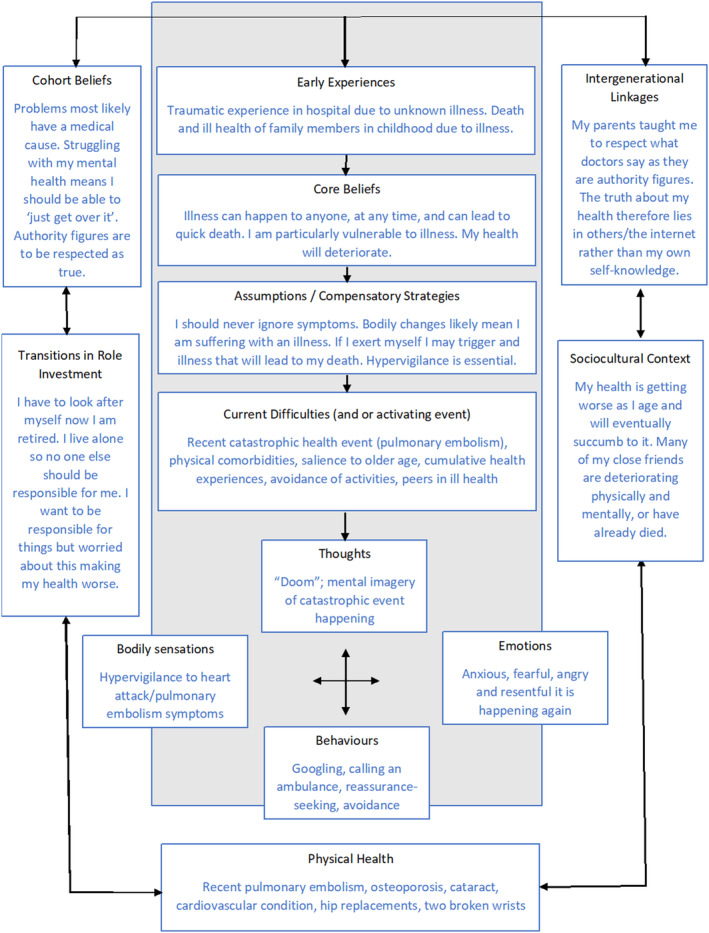
Longitudinal formulation of Evie's health anxiety, adapted from Laidlaw.^18^

A second formulation captures contextual, biological, sociocultural, and psychological elements of Evie's health anxiety from a longitudinal perspective. This formulation derives from the model of older adult CBT by Laidlaw et al.^18^, ^19^ and is presented in Figure 2. This formulation adds the contributory role of the traumatic health‐related experiences, cumulative experiences of death and ill health, of Evie's upbringing. Through guided discovery, aspects of Evie's life related increasing identification with older age, namely by role transitions, physical/mental health, social network, cohort beliefs, and intergenerational linkages, were incorporated into the formulation.

### 4.3 Outcome measurement: HAI; GAD‐7; PHQ‐9; WSAS

**FIGURE 3:**
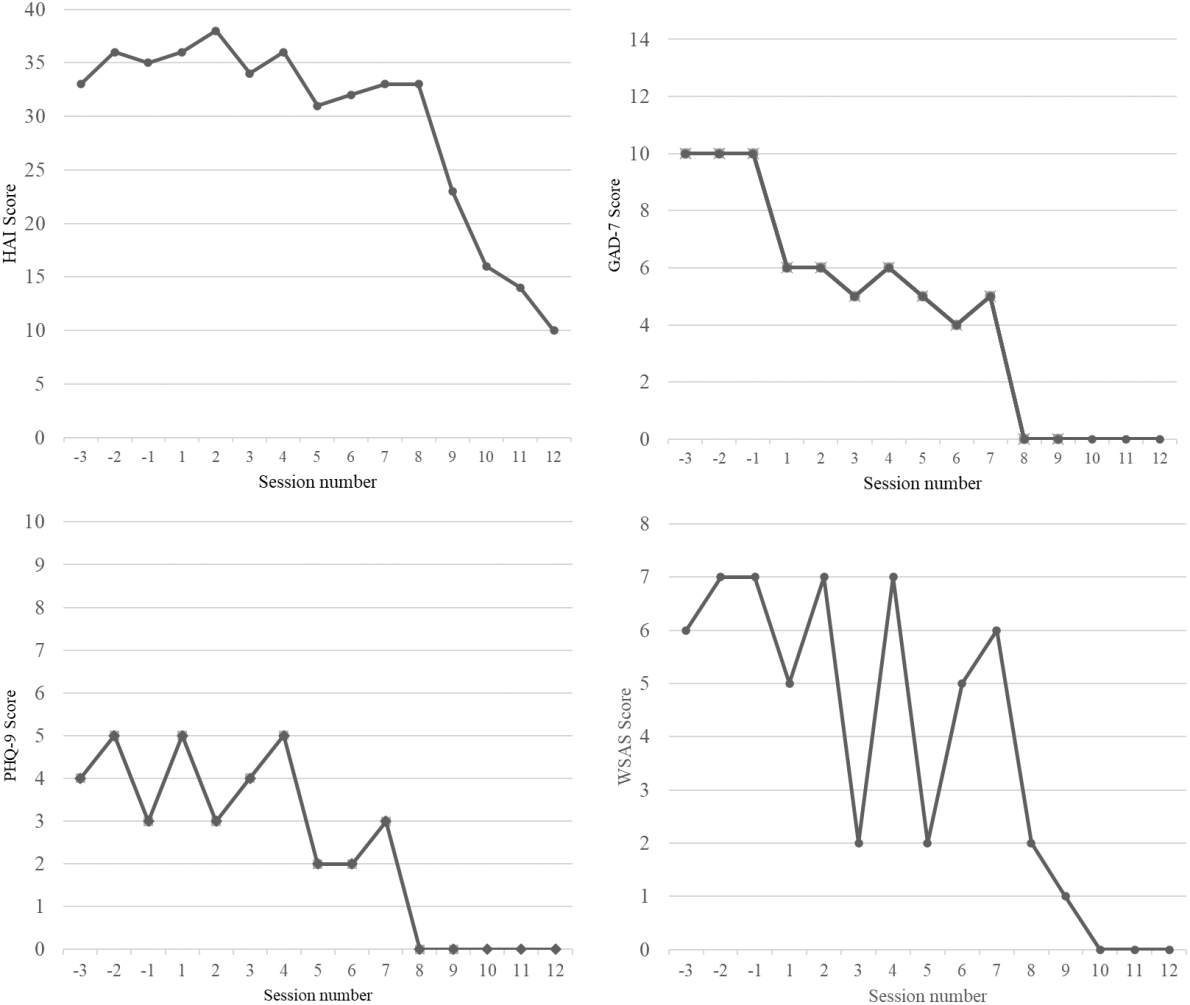
Evie's HAI, GAD‐7, PHQ‐9, and WSAS scores over the course of treatment and 3 weeks prior to treatment.

Over the treatment course, Evie's HAI scores reduced from 36 to 15; Evie's GAD‐7 scores reduced from 6 to 0; Evie's PHQ‐9 scores reduced from 5 to 0, and Evie's WSAS scores reduced from 6 to 0 (Figure 3).

### 4.4 Symptom googling

**FIGURE 4:**
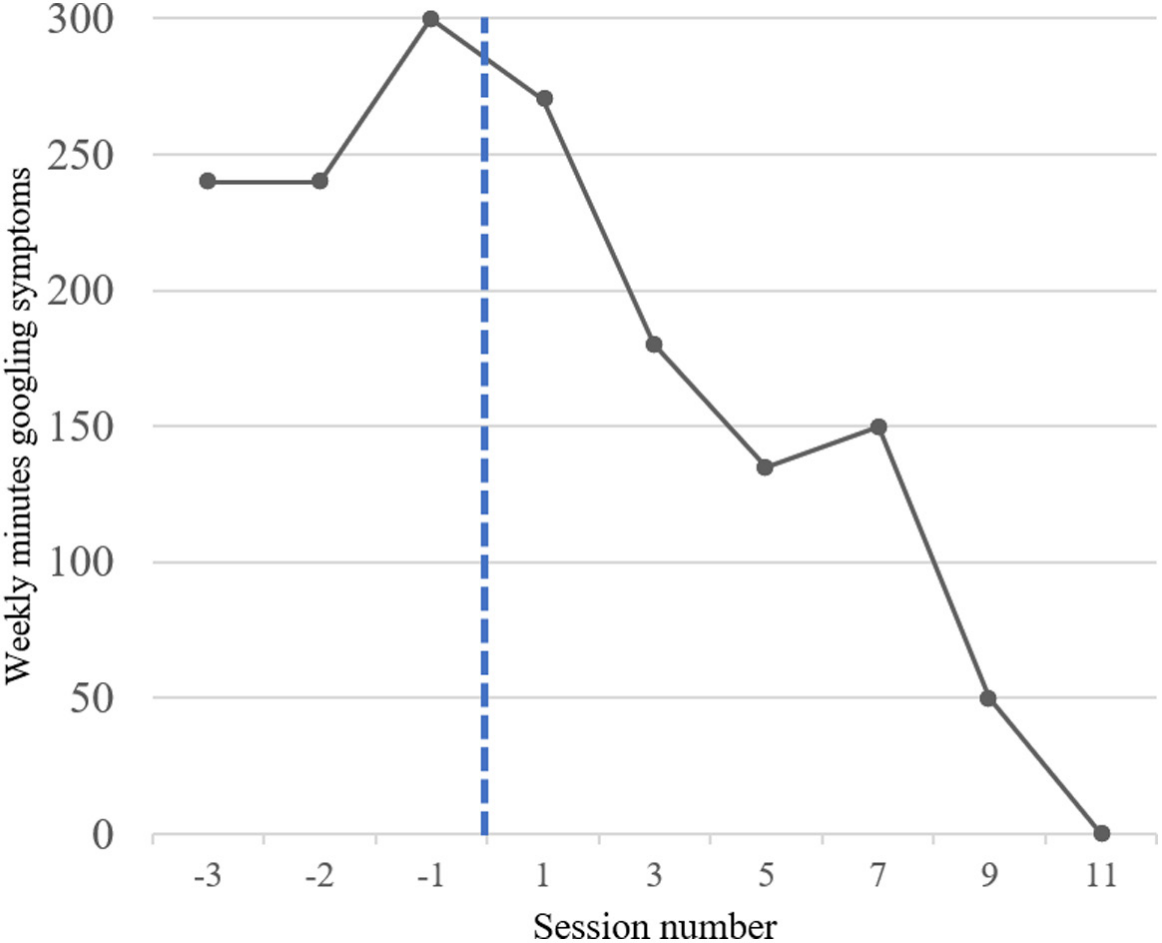
Evie's self‐reported weekly minutes googling symptoms 3 weeks prior to treatment and biweekly throughout. A dashed line indicates pre‐intervention (Phase A, left of the line) and intervention (Phase B, right of the line) stages of measurement.

Visual examination of Figure 4 appeared that the intervention likely contributed to reductions in symptom googling. Before treatment (Phase A), Evie presented with a baseline of 240–300 weekly minutes googling symptoms. This declined over the course of Phase B, to 0 min per week at Session 11. The pattern of reduction in Phase B appears to mirror the trendline of health anxiety symptom reduction (Figure 3) but roughly 2–3 sessions ahead of HAI reductions. At follow‐up, Evie reported 0 weekly minutes googling symptoms, indicating a sustained reduction in googling over a 3‐month period.

### 4.5 Overall therapy progress and achievement of goals

Sessions 10–12 involved collaborative reviews of Evie's four treatment goals. With respect to these goals, the following were established: (1) All measured psychometric scores reduced to below clinical thresholds; (2) Evie was regularly participating in activities she had previously avoided due to health‐related worry, including regular social events, practicing music, and tidying her house; (3) Evie reported finding her soothe box and other behavioral interventions useful during times of heightened anxiety; (4) Evie showed a marked reduction in weekly googling: on Session 12, she reported “*I haven't felt the need for googling for some time now*.” These suggest an overall achievement of goals. On a subjective level, Evie reported feeling *“far less self‐limiting than [she] was”* and a keenness to maintain progress.

## 5 DISCUSSION

The present study appears to demonstrate the effectiveness of CBT for health anxiety using the protocol by Salkovskis et al.^17^ in the context of older age. This is evident through a reduction in HAI, PHQ‐9, GAD‐7, and WSAS scores. In addition, the single case experimental design component of this study demonstrates the effectiveness of CBT for health anxiety in reducing maladaptive health‐related googling, which appeared to be sustained 3 months post‐intervention.

On a subjective level, Evie reported that increasing her daily activity reduced the time she had to engage in the rumination which precipitated symptom googling. As such, activity scheduling and behavior activation may be effective indirect treatments for maladaptive googling, wherein the function appears to supplement rumination. She additionally reported the grounding strategies provided were usually sufficient to prevent ruminative thoughts leading to googling.

When using CBT for older adults, it is important that issues pertinent to older age, namely health conditions, cohort beliefs, intergenerational linkages, and role transitions, are attuned to. This was highlighted in three main ways during Evie's treatment. First, Evie's health‐related beliefs were rooted in traumatic health‐related experiences in childhood, which interacted with the increased salience of mortality and ill health in older age. Second, her perception of becoming increasingly weak, together with views that physical exertion has a high probability of catastrophic outcomes, led to catastrophic worry about physical activity, and high avoidance of important life activities. Evie responded well to interventions aimed at reducing avoidance, however if her beliefs led to marked resistance to engaging in these exercises, in vivo behavioral experiments testing beliefs extracted from “Theory A/Theory B” exercises, would have been implemented to facilitate engagement. Thirdly, her cohort beliefs regarding acceptance of information from authority figures appeared to increase her belief in health information gained from googling.

### 5.1 Clinical implications

This study contributes to the evidence that CBT for health anxiety is effective in older adult populations, and that incorporating the contributory role of symptom googling may benefit such treatment. The intervention likely contributed to reductions in symptom googling which were sustained at follow‐up, substantiated by subjective reporting from the client of no longer experiencing thoughts to google health information. Evie engaged readily in all stages of the intervention, with all psychometrics scoring in the subclinical ranges at the end of 12 sessions. This study also punctuates the importance of googling as a specific maintenance variable within some cases of health anxiety that should be treated accordingly.

Despite this, our understanding of googling within health anxiety frameworks is limited, and although googling likely serves heterogeneous functions across cases, this case study offers an anecdotal account which could direct research into its understanding. Evie's case supports the notion that googling can serve a parallel function to rumination, with the additional function of providing further information the individual unduly interprets as confirming their health suspicions. In cases such as Evie, who displayed cohort beliefs of perceiving authority figures (which can include Google) as correct, this may be especially pertinent. Further clinical reports detailing maladaptive googling experiences will further the literature base of this phenomenon.

Clinicians working with clients exhibiting maladaptive googling may consider strategies intended to disrupt rumination, such as grounding and rapid problem‐solving exercises, incorporating psychoeducation regarding reassurance‐seeking, and perceiving online information as true. This case study punctuates the importance of understanding the function of the googling, when considering targeted interventions.

### 5.2 Study limitations

The coincidental reduction in health anxiety symptoms and symptom googling make it unclear as to the interaction, both in terms of direction and mechanisms, between googling, and health anxiety. For example, it is unclear whether reducing googling led to improvements in health anxiety, or if aspects of the CBT reduced the googling. The reduction in googling appears to follow a similar trendline to HAI reduction, but roughly 2–3 sessions ahead, which may weakly suggest that reducing googling may engender reductions in health anxiety. It should be noted that this was concluded in part through visual graph inspection which lacks specificity on the level of improvement. The nature of symptom googling in health anxiety is poorly understood and, due to the potential dangers it can engender in these populations, in critical need of exploration.

Although behavioral interventions for elevating mood were introduced to facilitate reduction in health anxiety symptoms, Evie's PHQ‐9 scores were below clinical threshold at baseline and throughout treatment, which eased engagement in behavioral techniques. As such, it is unclear whether the pattern of reduction in health anxiety symptoms Evie demonstrated would be expected in cases of higher initial depression severity.

## AUTHOR CONTRIBUTIONS

**Sean Hill:** Conceptualization; methodology; project administration; writing – original draft; writing – review and editing. **Daniel Watts:** Conceptualization; methodology; resources; supervision; writing – review and editing.

## FUNDING INFORMATION

This research received no specific grant from any funding agency, commercial or not‐for‐profit sectors.

## CONFLICT OF INTEREST STATEMENT

The authors declare no conflict of interest.

## ETHICS STATEMENT

All authors have abided by the Ethical Principles of Psychologists and Code of Conduct as set out by the British Association for Behavioral and Cognitive Psychotherapies (BABCP) and the British Psychological Society (BPS). The person described has seen the submission in full and agreed to it going forward for publication.

## CONSENT

Written inform consent was obtained from the patient to publish this report in accordance with the journal's patient consent policy.

## Data Availability

The authors confirm that the data supporting the findings of this study are available within the article.
